# Porous Aerogels and Adsorption of Pollutants from Water and Air: A Review

**DOI:** 10.3390/molecules26154440

**Published:** 2021-07-23

**Authors:** Paola Franco, Stefano Cardea, Antonio Tabernero, Iolanda De Marco

**Affiliations:** 1 Department of Industrial Engineering, University of Salerno, Via Giovanni Paolo II, 132, 84084 Fisciano, Italy; pfranco@unisa.it (P.F.); scardea@unisa.it (S.C.); 2 Department of Chemical Engineering, University of Salamanca, Plaza los Caídos s/n, 37008 Salamanca, Spain; 3 Research Centre for Biomaterials BIONAM, University of Salerno, Via Giovanni Paolo II, 132, 84084 Fisciano, Italy

**Keywords:** porous aerogels, adsorption, pollutants, environment, organic dye removal, wastewater, VOCs

## Abstract

Aerogels are open, three-dimensional, porous materials characterized by outstanding properties, such as low density, high porosity, and high surface area. They have been used in various fields as adsorbents, catalysts, materials for thermal insulation, or matrices for drug delivery. Aerogels have been successfully used for environmental applications to eliminate toxic and harmful substances—such as metal ions or organic dyes—contained in wastewater, and pollutants—including aromatic or oxygenated volatile organic compounds (VOCs)—contained in the air. This updated review on the use of different aerogels—for instance, graphene oxide-, cellulose-, chitosan-, and silica-based aerogels—provides information on their various applications in removing pollutants, the results obtained, and potential future developments.

## 1. Introduction

Aerogels are a particular class of three-dimensional materials characterized by an open, highly porous, and air-filled structure [1] with low density and thermal conductivity, a high degree of porosity, and an enormous specific surface area [2].

A wide variety of materials—including organic, inorganic, or hybrid molecular precursors—allow the fabrication of aerogels, with the possibility of obtaining different morphologies/shapes of various dimensions and pore size distributions [3,4,5].

In addition to aerogels based on a single material, the production of composites consisting of different materials is of interest in presenting the chance to tailor and improve the properties of aerogels, such as water-affinity, mechanical resistance, and their performance for specific applications [6,7,8].

Because of these peculiar properties, aerogels are attractive for a wide range of applications, from drug delivery [9,10] to packaging [11,12], from thermal insulation [13,14] to energy storage [15,16] or catalysis [17,18]. In addition, due to their excellent adsorption capacity, aerogels emerged as superior adsorbents for the removal of contaminants contained in both water and air [19]. Indeed, in the current scenario of environmental remediation, the purification of air and wastewater is still a challenge. Specifically, aerogels have been successfully used to remove various categories of pollutants, such as pharmaceuticals [8,20,21], dyes [22,23], oils and solvents [24,25,26], heavy metals, and radioactive elements [27,28,29]. The presence of these organic compounds in water and air—even in small quantities—poses a severe danger to all living organisms and the environment.

Thus far, different kinds of aerogels have been proposed for air and water treatments; however, those based on cellulose, chitosan, graphene oxide, and silica are among the most commonly employed [17,19,30,31,32]. Generally speaking, over the years, aerogels’ evolution began with the appearance of silica aerogels in the 1970s, followed by carbon-based aerogels; then, the novel aerogels of the 2010s were proposed, such as those based on graphene [19]. However, the interest in using biopolymer-based aerogels, such as chitosan and cellulose, is increasing, in order to reduce their environmental impact [2].

In some cases, continuous processes based on graphene oxide [4] or silica aerogels [33,34] were developed to remove pollutants from wastewater.

This updated review provides an overview of recent advances—particularly highlighting the past six years (from about 2016)—in the application of aerogels for air and water purification, which is a current hot topic. Specifically, our attention is focused on the different applications of cellulose-, chitosan-, graphene oxide-, and silica-based aerogels, providing solutions to remove various pollutants.

Furthermore, this review will also be valuable to identify strategies and methodologies based on surface engineering or aerogel preparation and composition, so as to tailor the properties of the aerogels depending on their final application. In this context, the selected materials are adequate for this purpose, since they have different origins, and can even be combined to produce hybrid materials with a wide range of properties.

## 2. Cellulose-Based Aerogels

### 2.1. Synthesis of Cellulose Aerogels

Among the various materials used for environmental remediation, cellulose-derivate aerogels are among the most promising and studied. Their potential applications range from air cleaning (such as adsorption of volatile organic compounds) to water treatment processes (such as adsorption of oils, dyes, and hazardous organic compounds). Indeed, cellulose is an economical and abundant material extracted from several sources, such as plants and plant-based materials (rice, cotton, wood, etc.) [31]. Moreover, the preparation of aerogels from cellulose materials is convenient because the cellulose chain is rich in hydroxyl groups, so no crosslinking agent is needed in the aerogel preparation process. This means that a stable, three-dimensional network structure can be obtained via the intramolecular and intermolecular physical crosslinking of hydrogen bonds, thus making the aerogel preparation process relatively simple. Then, the chemical modification of cellulose to improve the mechanical strength and structural characteristics (from hydrophilic to hydrophobic) of cellulose aerogels is relatively easy to accomplish [35,36,37,38].

The preparation method and structural properties of cellulose aerogels are primarily dependent on the performance of cellulose and its concentration [31]. Therefore, cellulose aerogels are divided into three categories based on their raw materials: natural cellulose aerogels (nanocellulose aerogels, bacterial cellulose aerogels), regenerated cellulose aerogels, and cellulose-derivate aerogels. The classical sol–gel process allows the generation of the gel, but it varies based on the particular type of cellulose aerogel desired. For example, because the molecular chains of cellulose derivatives have a reduced number of hydroxyl groups, a crosslinking agent is generally needed in order to obtain a stable gel structure. Regenerated cellulose gel is prepared by the regeneration of cellulose solutions, whereas nanocellulose gel is made from a nanocellulose suspension.

The drying of the cellulose-derivate gel, and the consequent generation of the aerogel, is known as it is the most critical step of the process. Substantially, two kinds of drying methods have been successfully tested on cellulose-derivate gels: freeze-drying [39,40,41], and supercritical gel drying [42,43,44]. Generally speaking, aerogels prepared by drying with supercritical fluids usually present a cauliflower-like arrangement of cellulose: an agglomeration of tiny, shaggy beads. However, freeze-drying leads to a sheet-like cellulose network, with large and interconnected pores that are several micrometers in diameter, due to ice growth during water freezing [45].

Considering the environmental remediation applications, several authors have proposed using cellulose-derivate aerogels alone or in composite structures. Some of the most interesting and recent works are reported in Table 1.

### 2.2. Application of Cellulose-Based Aerogels

Observing Table 1, it is evident that cellulose-derived aerogels are mainly used to remove oils and solvents. In addition, in some works, they are used to remove dyes, in a couple of papers as gas adsorbents, and in one article to remove metals.

Concerning the use of cellulose-based aerogels to adsorb gases, Gebald et al. [46] proposed the generation of a nanofibrillated cellulose (NFC) hydrogel functionalized by amine to be used as an adsorbent for the capture of CO_2_ from air. The aerogel’s morphology was characterized by cellulose sheet structures, caused by the presence of the amine, with single distributed cellulose nanofibrils; BET analyses indicated a surface area of 7.1 m^2^/g, with an amine loading of 4.9 mmol N/g. Comparing the surface area value with that of the NCF aerogel (without amine), it is evident that amine’s addition caused a decrease in surface area from 26.8 m^2^/g to 7.1 m^2^/g. Regarding the adsorption of CO_2_ in air, at a CO_2_ concentration of 506 ppm in the air, at 25 °C, 1.39 mmol CO_2_/g was adsorbed, confirming the capability of NFC aerogels to work as good adsorbents.

In a recent paper, Kiliyankil et al. [6] removed odorous gases (i.e., ammonia, hydrogen sulfide, methyl mercaptan, trimethylamine) from the air, using NFC-based aerogels loaded with nanoparticles (NPs) of metal compounds (such as copper, cobalt, and nickel). The NFC-based aerogels were obtained via a freeze-drying method, starting from NFC gels loaded with metal nanoparticles; carbon nanotubes (CNTs) were also incorporated in the NFC aerogels, in order to increase their surface area and improve their mechanical characteristics. The final aerogels possessed very low densities (5–10 mg/cm^3^), very high porosities (up to 99.17%), and micrometric and nanometric pores. The authors justified the low aerogel surface area (10 m^2^/g) with the presence of large pores in the aerogel, which were not included in the BET surface area analysis. Regarding the adsorption experiments, the authors tested different metals loaded in NFC-CNT aerogels on various gases; for example, copper-loaded aerogels were tested on ammonia (150 ppm in air), showing high performance, with 95% removal in 10 min, and more than 99% in 30 min. Similar results were obtained for trimethylamine (70 ppm in air), methyl mercaptan (100 ppm in air), and hydrogen sulfide (20 ppm in air) adsorption: 97% trimethylamine removal after 1.5 h, 100% methyl mercaptan removal after 1.5 h, and 100% hydrogen sulfide removal after 10 min.

Given the encouraging results shown in the two papers just discussed, the use of NC-based aerogels as gas adsorbents for air purification should be deepened, and research in this field encouraged.

Another exciting field where the cellulose-based aerogels were successfully tested is in the removal of oils, solvents, and dyes from water. Of course, to properly use cellulose-based aerogels in oil removal applications, it is necessary to convert the inherent hydrophilicity of aerogels to hydrophobicity and oleophilicity, in order to work with high oil/water selectivity.

Sai et al. [47] prepared bacterial cellulose aerogels (BCAs) via a freeze-drying process, and used trimethylchlorosilane and triethylamine as the modifying agents to make the BCAs hydrophobic. The aerogels presented a nanofibrous structure, with diameters ranging between 20 and 80 nm; the porosity was higher than 99%, and the surface area higher than 169 m^2^/g. The authors investigated the absorption performance of the aerogels for different oils and solvents, such as gasoline, diesel, toluene, plant oil, paraffin, acetone, etc. The results were encouraging for all of the substances tested. Indeed, the aerogels showed high mass adsorption capacity: up to 185 g/g. He et al. [25] prepared a composite aerogel with bacterial cellulose (BC) and SiO_2_ to generate a superelastic and superhydrophobic structure for the adsorption and recovery of oil from water. First, a BC hydrogel was prepared and freeze-dried in order to obtain an aerogel; then, a SiO_2_ solution impregnated the BC aerogel, producing a BC-SiO_2_ gel. Finally, the composite gel, after the solvent exchange in ethanol for 24 h, was freeze-dried. The final structure was characterized by a nanofibrous network (due to BC), with fibers of about 50–100 nm, overlapped to a 3D porous matrix, which assured an elastic behavior. The composites presented a hierarchical cellular structure, including macropores due to BC fibers and mesopores due to silica aerogels. This combination ensured the formation of a superelastic system that can bear a compressive strain up to 80%, with a complete recovery of the original volume after the stress release. Moreover, the final aerogel was hydrophobic, with a contact angle value of about 152°. Regarding the oil adsorption, the BC-SiO_2_ aerogels were tested against different water solutions containing ethanol, dimethylformamide, pump oil, motor oil, gasoline, and plant oil. In all cases, the aerogels showed very high performances, and oil recovery of about 88% from aerogels was also possible. 

Different groups proposed the generation and use of a recycled cellulose aerogel (RCA) through freeze-drying for oil absorption. Feng et al. [48] and Nguyen et al. [49] coated the final products with methyltrimethoxysilane via a chemical vapor deposition step in order to create superhydrophobicity. In the former paper [48], the authors obtained a porous structure characterized by macropores larger than 50 nm, with contact angles ranging between about 150 and 153°. Then, oil absorption capability was investigated using motor oils: the absorption capacity varied between 49 and 95 g/g, depending on the cellulose concentration; it was one order greater than those of the natural sorbents and two to four times greater than those of the commercial sorbents. In the latter paper, Nguyen et al. [49] tested three different solutions containing three crude oils to study the absorption efficiency of their RCA: the maximum absorption capacities at ambient conditions ranged between 18.4 and 20.5 g/g—double those obtained with traditional absorbents for crude oils. 

Han et al. [58] used waste newspaper to obtain RCAs. Aerogels were obtained via a combined process of freeze-drying and pyrolysis; SEM images showed a porous and interconnected 3D fibrous network, with fibers of about 3.7 µm. The water contact angle was about 132°, confirming the hydrophobicity of the aerogels. The absorption capacity of the generated aerogels was tested against different oils (pump oil, gasoline, olive oil, etc.) and solvents (ethyl acetate, chloroform, benzene, acetone, ethanol, etc.) in water. The aerogels showed a high absorption capacity for all of the oils and organic solvents tested, with 16–26 times higher absorption capacities than the raw material (i.e., waste newspaper).

Lin et al. [50] proposed a simple cellulose aerogel as an adsorbent. Analyzing SEM images, a woven, porous, three-dimensional network was evident (fibers of about 20 µm), with a porosity value of up to 98.7%. Additionally, in this case, the aerogels were made hydrophobic and oleophilic; in particular, the authors proposed a surface modification via cold plasma technology, and obtained surfaces with a contact angle of 150°. The samples were tested as absorbents against different oils, and showed a higher adsorption capacity than the traditional adsorbents.

Nanocellulose (NC) aerogels were used by different groups for several applications, using different ways to functionalize NC to obtain hydrophobic and oleophilic aerogels. For example, Wang et al. [51] tested them for superabsorbence, flocculation, and oil-water separation. Different kinds of pores (from mesopores to macropores), with an overall porosity up to 98%, were detected; moreover, a specific surface area ranging from about 85 to 147 m^2^/g was measured. The samples showed very high coagulation–flocculation capability to treat wastewater with CaCl_2_ (87.1%), increased removal efficiency of dye uptake (127.73 mg/g), and good Cu^2+^ absorption capacity (45.053 mg/g).

Khoronen et al. [52] functionalized nanocellulose aerogels (NCAs) with a nanometric layer of titanium oxide (TiO_2_), using an atomic layer deposition step to increase the samples’ hydrophobicity and oleophilicity. NCAs were tested as absorbents against paraffin oil and mineral oil: the final absorption was close to the overall volume of the aerogel (up to 90% vol/vol), and the mass-based absorption capacity varied from 20 to 40 (wt/wt), depending on the density of the liquid. Moreover, the authors showed that the NCAs’ absorption capacity was not modified upon repeated immersion and drying. Thus, the aerogels were reusable, which makes them suitable for the required applications. 

Zhou et al. [24] used methyltriethoxysilane to obtain NCA surface modification. Through SEM images, the authors observed the presence of nanofibers aggregated into porous or sheet-like structures, for NCAs both before and after the silanization step. This result indicates that the silanization treatment did not affect the absorbent structures of the original aerogels. The samples presented very high porosity (from 99.68 to 99.79) and surface areas (from about 95 to 196 m^2^/g). The hydrophobicity of the final aerogels was confirmed via the measurement of contact angles (values up to about 152° were found). The absorption capacities of NCAs were tested against various oils and organic solvents in water: high absorption capacities were found for all of the oils and organic solvents tested (i.e., up to 159 g/g for oil and 260 g/g for chloroform). The authors also proposed an interesting comparison between their NCAs and other absorbents: for example, with synthetic polymers (14–57 g/g), cellulose fibers (20–50 g/g), nanocellulose-based absorbents (20–185 g/g), chitin (29–58 g/g), carbon aerogels (29–192 g/g), and silica aerogels (16 g/g); for all cases, the NCAs generated in this paper presented a higher absorption capacity. The authors also studied the reusability of NCAs, finding that the adsorption capacity still exceeded 92 g/g for pump oil after 30 absorption cycles. Jiang et al. [53] treated NCAs with triethoxyl(octyl)silane to convert them from hydrophilic to hydrophobic and oleophilic. NCAs were able to absorb 200–375 times more non-polar hydrocarbons, polar solvents, and oils; for example, they absorbed 187 g/g of acetone, 268 g/g of DMSO, 356 g/g of chloroform, and 219 g/g of decane. Moreover, the absorbed liquids can be easily distilled and recovered.

Hasan et al. [54] adopted silane to obtain hydrophobic structures, using the aerogels for the removal of dyes from water; the silane modification was performed following the same procedure reported by Zhou et al. [24]. Again, SEM analysis allowed them to evidence porous and sheet-like structures for all aerogels obtained. Moreover, polysiloxane particles were visible on the surface of the NCA after the silane modification, which resulted in the hydrophobicity of the fabricated silane-modified NCAs. The absorption capacity of the NCAs was tested against crystal violet dye, and absorption of 150 mg/g was found after 2 h. The authors also analyzed the mechanical resistance of the materials, comparing the pure aerogels with the silane-modified aerogels. The former presented a compressive modulus of about 102 KPa, the latter a compressive modulus of about 210 KPa; this result was explained considering the presence of crosslinked Si-O-Si bonds of polysiloxane.

NFC-based aerogels have been used in composites. For example, Wu et al. [55] incorporated NFC with graphene oxide (GO) platelets in the same aerogel, Lyu et al. [22] prepared NFC-based aerogels combined with polyaniline (PANI), whereas Ji et al. [57] functionalized NFC with tannic acid (TA). Varying the concentration of NFC in the aerogels constituted by NFC and GO, surface areas ranged from 128 to 581 m^2^/g. The authors analyzed the adsorption performances of the aerogels with anionic methyl orange, cationic rhodamine 6G, and silicon oil solutions. They showed that the presence of NFC at a suitable concentration can improve both hydrophilicity and hydrophobicity, and so can enhance the affinity of the final composite aerogel toward the adsorption of not only hydrophilic substances, but also hydrophobic organic oil. The NFC-PANI aerogels were tested as absorbents against acid red and methylene blue. They showed highly efficient adsorption capacity for acid red (i.e., about 600 mg/g) and methylene blue (about 1363 mg/g) via π-π stacking electrostatic interaction. Moreover, multiple regeneration experiments showed that NFC-PANI aerogels could maintain high adsorption capacity (about 84% for acid red and 70% for methylene blue) after three regeneration cycles. NFC-TA aerogels were used to capture Cu(II) and organic contamination. SEM images showed the typical morphology of NFC aerogels with different surface areas, depending on the presence of TA and on the deposition of CDA (i.e., from about 76 to 151 m^2^/g). Moreover, outstanding mass absorption capacities versus organic pollution (for example, up to 108 g/g for chloroform) were obtained.

In a single paper, the supercritical drying was compared to freeze-drying. Indeed, Wang et al. [56] studied the formation of cellulose laurate ester (CE) aerogels and their behavior as absorbents of pyridine (polar solvent) and chlorobenzene (non-polar solvent) in simulated organic wastewater via a batch static absorption process. Analyzing SEM images, the aerogels obtained through both the drying techniques were characterized by a 3D, highly porous network with large interstitial spaces. However, a significant improvement in the specific surface area (SSA) was observed for the supercritical carbon dioxide (scCO_2_) dried gels (SSA: 152 m^2^/g), compared to the freeze-dried gels (SSA: 105 m^2^/g). The authors tried to explain this result, considering the partial collapse of the structure caused by the freeze-drying step; indeed, it is known that scCO_2_ allows function without surface tension. 

For this reason, scCO_2_-dried samples showed a more homogeneous structure, with more uniform, nanometric pore structures, compared to the freeze-dried samples. These results confirmed that scCO_2_ drying is the best method for preserving the gel structure after the drying step. Regarding the absorbent capacity, CE aerogels showed excellent absorption performance for both pyridine and chlorobenzene, with maximum absorption capacities of 9.63 mmol/g for pyridine and 18.38 mmol/g for chlorobenzene. Comparing the scCO_2_-dried aerogels with freeze-dried aerogels for chlorobenzene absorption, the first showed a maximum absorption capacity of 18.38 mmol/g. In contrast, the second showed a maximum absorption capacity of 13.60 mmol/g, confirming the better performance of scCO_2_-dried aerogels.

## 3. Chitosan-Based Aerogels

### 3.1. Synthesis of Chitosan-Based Aerogels

**Figure 1:**
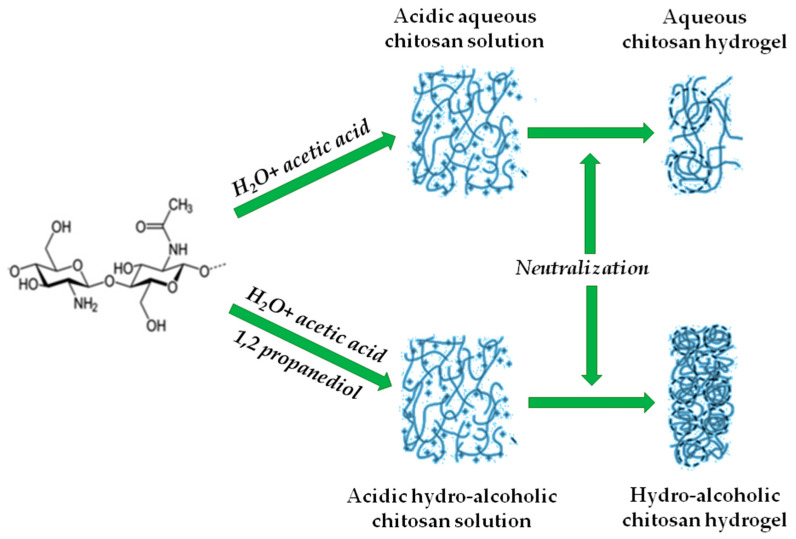
Steps to produce physical chitosan gels.

Chitosan is a cationic polysaccharide, from the glycosaminoglycan family, with a backbone of beta(1→4)-linked glucosamine and N-acetyl-glucosamine (Figure 1). Chitosan and its derivatives are biodegradable, biocompatible, nontoxic, and environmentally friendly polymers [59].

Chitosan is only soluble in acidic water after NH_2_ protonation. This phenomenon allows a physical gelation of chitosan (after polymer solubilization in water) by increasing the pH of the solution (with NaOH, for instance), promoting a sol-gel transition due to intermolecular interactions (Figure 1). Physical gels of chitosan can also be obtained via different means, since β-glycerol phosphate and temperature can also produce physical gels [60].

Chitosan chemical gels can also be produced using different crosslinkers, due to the functional groups of the polymeric chemical structure. Glutaraldehyde, genipin, and epichlorohydrin are commonly used for this purpose due to their ability to form bonds with amino groups. Moreover, it is also possible to produce a gel via electrostatic interactions between chitosan and different compounds, such as alginate or graphene oxide. Detailed information about chitosan hydrogel preparation can be found elsewhere [59].

Chitosan aerogels can be obtained after drying the physical or chemical gels with different techniques (e.g., supercritical drying or freeze-drying). Techniques such as ice templating—which consists of formatting ice crystals inside the structure of the previous gel [61]—or directional freeze-drying to obtain an anisotropic aerogel, can also be used for that purpose [62].

### 3.2. Applications of Chitosan-Based Aerogels

Concerning the potential of chitosan aerogels for removing pollutants, it is essential to highlight that their structure, with amino and hydroxyl groups, as well as the typical high surface area of aerogels, are helpful in adsorbing dyes (mainly with a negative charge), heavy anions, radionucleotides, and even carbon dioxide. Moreover, chitosan can chelate metals, and its functional groups can be chemically modified to synthesize carboxymethyl chitosan [63], or even a polymer with quaternary ammonium groups [64].

However, some drawbacks have to be considered before using chitosan aerogels for the removal of pollutants—mainly, mechanical resistance, and water stability. In general, aerogels’ Young’s modulus is usually low, and chitosan tends to hydrolyze in acidic water, whereas this polymer is not soluble in water at a neutral pH. Finally, the methodology to produce the hydrogel (physical or chemical), along with the drying technique, must be carefully chosen, since a certain crosslinker can reduce the active sites. In this context, epichlorohydrin does not block the amino groups. It can be a perfect crosslinker for heavy metal adsorption [65], whereas the physical gelation can produce gels with lower mechanical resistance and water stability [66]. Furthermore, the adsorption rate can be decreased due to the disorder in the network, which can be improved by controlling the microstructure with a directional drying method [62]. 

For the reasons mentioned above, it is not expected to use chitosan aerogels alone to remove pollutants. Chitosan usually acts as the main backbone, or as a secondary material, in a composite to improve aerogel properties, depending on the application. 

Table 2 shows many aerogels, mainly produced in recent years (from 2017), constituted by chitosan or its derivatives. This table also indicates the used drying technique, aerogel surface area, application, and adsorption efficiency.

#### 3.2.1. Chitosan Aerogels for Water Purification

Based on Table 2, it is possible to observe how chitosan-based aerogels have been developed for water purification, removing heavy metals—such as Pb(II), Cu(II), Cr(VI), and Cd(II)—and radionucleotides (U(VI)). Other applications can be for dye adsorption and oil/water purification. The reason for this was explained previously, and is related to the chitosan functional groups and the aerogels’ surface area.

Concerning the use of composites for the purposes mentioned above, chitosan has mainly been used with GO and cellulose. Chitosan can form stable aerogels with GO due to electrostatic interactions or chemical functionalization. The use of chitosan in this composite is beneficial, since it avoids the problems of GO recovery after water purification (high-speed centrifugation is needed, which is a critical drawback when working with a high volume of fluid). Moreover, although GO is added to increase the surface area and improve chitosan’s stability in acidic water, it is crucial to consider that an overstacking phenomenon of graphene sheets can involve a surface area loss in the drying step. For that reason, an aerogel formulation is required. In this context, Yu et al. [73] and De Luna et al. [74] included GO in chitosan aerogels, increasing the adsorption of anionic dyes (indigo carmine 524 mg/g), cationic dyes (methylene blue 168.6 mg/g), and Cu^2+^ (25.4 mg/g).

Another compound that can often be found in composites is polydopamine (sometimes together with the GO). This compound provides the composite with additional active sites to increase adsorption, promote electrostatic interactions, or even as reaction sites with aldehyde or thiol groups (Michael addition or Schiff base reactions) to control the material’s hydrophobicity. This type of composite has been proposed to remove organic dyes, heavy metals, and even radionucleotides. That fact can be observed in the table, where an adsorption of 374.4 mg/g, 441.2 mg/g, and 415.9 mg/g was found for Cr^6+^ [68], Pb^2+^ [68], and U^6+^ [98], respectively. The addition of reduced GO and polydopamine can also be useful for developing superhydrophobic materials, conferring the chitosan aerogels with adequate properties for oil/water separation (Cao et al. obtained an efficiency higher than 90% [87]).

Other compounds commonly found in chitosan aerogels include cellulose and its derivatives. These compounds are mainly used to enhance the aerogels’ mechanical resistance due to the structure of cellulose, and provide a negative charge to the molecule in order to adsorb cationic dyes or water separation [78]. Bacterial cellulose [67], cellulose [65], cellulose nanofibers [78], or even waste paper [69] can be suitable for this purpose. However, the obtained composites can have low specific surface areas. For that reason, other approaches use additional materials to increase that value. In this sense, the use of metal–organic frameworks can increase the surface area multiple times. In this sense, 268.7 m^2^/g [67] and 457.75 m^2^/g [65] were obtained after adding two different metal–organic frameworks.

Although not profoundly studied, the selected drying technique is also essential. Freeze-drying is the chief method used to produce aerogels. However, this time-consuming technique has several disadvantages, such as the required time, or even problems maintaining the original hydrogel structure. The use of supercritical CO_2_ can overcome the previous drawbacks, promoting the formation of aerogels with a high surface area in a shorter time. Although supercritical drying has been widely used to obtain chitosan aerogels [104], only one article in the table used this technique to produce composite aerogels.

Another technique that was used, mainly with cellulose nanofibers, is directional freeze-drying. This drying step is adequate to form anisotropic aerogels with unique properties—primarily mechanical—and even for insulation [7]. This particular structure can also be obtained via an electrospinning process and a subsequent crosslinking process. This methodology was used to obtain a composite chitosan/GO aerogel that could remove different types of pollutants (dyes and heavy metals) with a faster equilibrium adsorption time [96].

More types of compounds, such as wastes or biomass, can be included to develop a new kind of chitosan composite. For example, the use of soot promotes the formation of a composite with enhanced mechanical resistance. It confers a higher adsorption value towards cationic dyes, while reducing the adsorption of anionic dyes (250 mg/g MB and 275 mg/g of indigo carmine) [82]. In contrast, the inclusion of waste paper (constituted by cellulose) also increased the mechanical resistance [69]. Finally, different kinds of biomass can be added to obtain unique properties (e.g., microalgae biomass to improve uranium adsorption up to a value of 571 mg/g [77]). Again, as happened with the GO, the use of a chitosan aerogel is required to avoid problems regarding biomass separation. More compounds, such as clays or different hydroxides, can also be included in the aerogels with other objectives, such as phosphorus removal (the addition of lanthanum hydroxide increased that value up to 148.33 mg/g [84]), or a surface area increase (237.4 m^2^/g) if silica was included in the chitosan aerogel [81].

There is another strategy to tune chitosan aerogels’ properties depending on their final intended application. Surface engineering has been proposed several times to modify chitosan’s structure. Carboxylic acid [63] or succinyl groups [99] increase polymers’ water solubility and stability, and confer the aerogel with adequate oil/water separation properties. An efficiency of 99% was found for succinyl chitosan aerogels for oil/water separation [99].

Previous paragraphs discussed different options that can be used to tune chitosan aerogel properties for removing pollutants. The used materials and their composition, the drying technique, and crosslinking methodologies must be considered in designing the best platform, depending on the final application. In this context, it is important to repeat that only one article in the table used supercritical CO_2_ to produce the aerogel. Supercritical drying has different advantages, since it can speed up the drying (from days to hours) without collapsing the structure or increasing the surface area. A comparison between different drying techniques to obtain chitosan aerogels, and the influence on pollutant adsorption, is missing. Such a comparison would provide important information concerning the best drying procedure for this issue. 

More strategies may be followed, and must be deeply explored. For instance, chitosan’s chelation ability can be an important advantage in binding metals to the aerogel. That fact can be helpful in synthesizing materials with magnetic properties (to improve the separation with a magnetic field) [63], or with better adsorption properties (binding titanium) [83]. Moreover, the addition of some materials can confer the aerogel with properties to react with some compounds and improve the final performance. As an example, an aerogel with MoS_2_ was successfully developed. This aerogel was used to enhance gold recovery, due to its ability to perform the previously required mineral reduction using light [94].

Therefore, the potential of chitosan aerogels in adsorbing pollutants from water is almost infinite. However, it is essential to realize that the process and the composite compounds must be carefully selected depending on the final application. Today, it is impossible to obtain an aerogel to remove a wide range of compounds.

#### 3.2.2. Chitosan Aerogels for CO_2_ Capture

The use of aerogels can provide several advantages compared to the conventional methodologies for capturing CO_2_. Specifically, problems such as corrosion, degradation of amines, and the production of toxic byproducts can be avoided. Chitosan can act as a correct platform for this type of compound capture, because the CO_2_ is attracted by amino groups and the high-density charge [105,106].

Moreover, the previously explained methodologies can also be used to improve chitosan aerogels’ abilities to remove CO_2_. Chitosan can be modified to obtain a structure similar to exchange resins [64]. The amino groups can also be modified to produce quaternary ammonium groups. The developed aerogel showed a potential application for capturing CO_2_ from 0.18 mmol/g, which is more than 35% higher than the conventional membranes.

Another strategy is the inclusion of other compounds inside the aerogel to increase the surface area. One such compound was a zeolite, which helped to increase the surface area up to 550 m^2^/g and enhance the mechanical resistance of the final aerogel [91]. This material was able to remove 4.23 mmol/g of CO_2_—far higher than the amount with chitosan/GO (0.26 mmol/g) [75].

The previous works indicated how chitosan aerogels could be used for CO_2_ capture. However, it is important to realize that these aerogels have to be developed with unique features, such as low water sorption and adequate thermal regeneration. With high versatility to be used in different devices at a large scale, these properties must be considered in a chitosan aerogel designed to capture CO_2_.

## 4. Graphene Oxide-Based Aerogels

### 4.1. Synthesis Methods for GO-Based Aerogels

**Figure 2:**
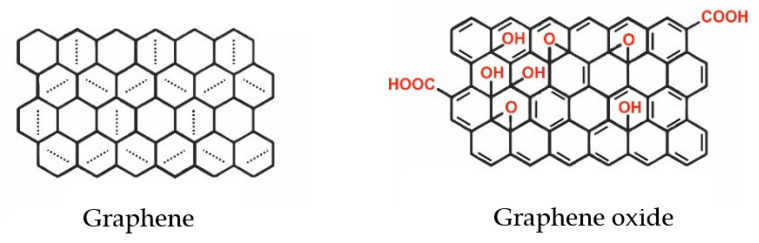
Structure of graphene and graphene oxide.

Graphene—a two-dimensional monolayer of carbon atoms forming a honeycomb lattice (Figure 2)—attracts considerable interest due to its excellent thermal stability, electrical conductivity, and physicochemical and mechanical properties [107,108]. Among various applications, graphene exhibits an incredible potential for water pollution control, specifically exploiting graphene oxide (GO) as an adsorbent to remove different kinds of pollutants from wastewater [5,109]. Although the surface area of GO (Figure 2) is smaller than that of graphene, the GO exhibits better stability than graphene, combined with good performance; thus, it is considered a better option than graphene [110].

GO is a cost-effective and nonconductive hydrophilic carbon material, which can be easily synthesized by the oxidation of the natural flake graphite powder. Today, GO is usually synthesized by Hummers’ method—one of the oldest techniques that involves the use of KMnO_4_ and NaNO_3_ in concentrated H_2_SO_4_ [111].

Hummers’ method is easily reproducible and scalable for large-scale production of GO by oxidizing graphite; however, there are some associated drawbacks, including the massive production of liquid, toxic waste. Hence, modern variations have been proposed over the years, known as modified Hummers’ methods. The aim is to improve the process—both in terms of efficiency, and from an environmental point of view—by changing the amounts of the original reagents, or replacing them with less dangerous alternatives that do not release toxic compounds [5,109,112].

The as-prepared GO can be reduced again by various chemical, thermal, electrochemical, or photocatalytic methods, obtaining reduced graphene oxide (rGO) [30]. The reduction process shifts from a functionalized and hydrophilic structure to an apolar and hydrophobic one.

Generally speaking, the GO’s large surface area and many oxygen-based functional groups (i.e., hydroxyl and epoxy groups on the basal planes; carbonyl and carboxyl groups at the sheet edges) are the key features. Indeed, they confer this material the ability to decontaminate wastewater [30,113].

However, the direct application of GO sheets for the removal of pollutants is limited by several factors: primarily, it tends to a layer-by-layer aggregation because of its strong planar interactions, leading to a possible decrease in its adsorption capacity; moreover, the oxygen-containing functional groups of GO are not very stable in coordination with pollutants [30].

In this context, GO has been widely used to synthesize graphene-based aerogels, since the oxygen portions can interact with different compounds—mainly biopolymers that can be covalently immobilized onto GO. In this way, new materials with enhanced biocompatibility and tailored properties for a specific application can be obtained [114].

GO-based aerogels are the most common 3D graphene structures, with extraordinary properties, such as lightness, excellent mechanical and thermal resistance, electrical conductivity, high surface area, and adsorption capacity [115].

**Figure 3:**
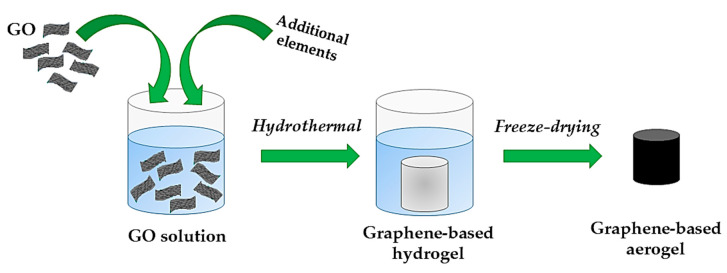
An exemplificative scheme of the GO-based aerogel’s synthesis via a hydrothermal method.

The main approaches employed to obtain GO-based aerogels are hydrothermal reduction/self-assembly (which exemplificative scheme is represented in Figure 3), chemical reduction methods, crosslinking, and sol-gel processes [110,114,116]. All of these procedures generally end with a drying step to obtain the final aerogel, with the most being common freeze-drying [28,117,118,119] or, to a lesser extent, supercritical drying [120].

However, the hydrothermal reduction method involves the self-assembly of the graphene sheets, working under high-temperature and high-pressure conditions [121,122,123]. On the other hand, it is also possible to conduct a chemical reduction method using mild reduction agents to restore the sp2 network [117]. Although it can be more interesting than the hydrothermal method—which requires high temperatures and pressures—the chemical reduction often leads to the attainment of a small surface area, because of the agglomeration of graphene layers due to π-π interactions [110].

The hydrophilic GO forms a stable solution in water; however, decreasing the pH of the GO solution reduces the electrostatic repulsion, and the hydrogen bond strengthens due to the protonation of carboxyls, leading to a stable GO gelation [114]. The gelation of GO sheets is triggered by crosslinking agents, strengthening the bonding force. Common GO crosslinkers are molecules that contain specific reactive groups, including hydroxyl, oxygen-containing, or nitrogen functional groups [3,28,117,118].

The sol-gel method is another route to synthesize graphene aerogels [119,124,125], in which the bonds between the GO sheets are stronger than those obtained via crosslinking. Specifically, in the sol-gel process, covalent bonds between the sheets are formed by polymerization [114].

Aside from the classic cylindrical structure, GO-based aerogels have been proposed in various forms, such as beads [3,126], microspheres [4], and flakes [5].

A summary of the general advantages and disadvantages of the manufacturing and use of GO-based aerogels is reported in Table 3.

### 4.2. Applications of GO-Based Aerogels

To the best of our knowledge, to date, GO-based aerogels or structures, in general, have been rarely employed for air purification. Specifically, in the study of Zou et al. [127], a GO membrane was proposed for the removal of PM_2.5_ particulate matter in the air. This GO membrane guaranteed high removal efficiency (up to 99.5%) for a long time.

On the other hand, graphene aerogels are largely and almost exclusively proposed to remove a wide variety of pollutants from wastewater, including dyes [128,129], drugs [8,20], heavy metals [121,130], radioactive elements [28,29], bacteria [124,131], organic solvents [131,132], and oils [26,125]. GO-based aerogels have exhibited great potential for wastewater treatment and the purification of natural waters, such as removing mercury [133] and microcontaminants from drinking water [134].

The study of Pan et al. [131] proved the versatility of GO/quaternary ammonium salt (QAS) aerogels in removing various categories of pollutants from wastewater. Indeed, organic dyes (methylene blue as a model), a wide variety of solvents (i.e., toluene, n-dodecane, cyclohexane, hexane, petroleum ether, dichloromethane, chloroform), and oils (i.e., gasoline, soybean oil) were removed. In addition, the GO/QAS aerogel exhibited a bactericidal effect, completely inactivating *Staphylococcus aureus* and *Escherichia coli* after a short contact time of 5 min. Moreover, the authors proposed a novel approach to prepare hybrid aerogels consisting of “spray-penetration-flocculation” to avoid uncontrolled precipitation of GO and the collapse of the structure during freeze-drying. For these purposes, QAS was selected as the crosslinking, flocculating, and antibacterial agent. The dispersion of QAS into the GO lattice simultaneously induced the in situ flocculation of GO, leading to the formation of a regular hydrogel network without the necessity of any additional steps. The as-fabricated aerogel exhibited a low density (i.e., ≤18.1 mg/cm^3^), a high porosity (i.e., 92–97%), and high adsorption capacities for the different pollutants. 

Similarly, Zhang et al. [124] loaded QSA into reduced GO/montmorillonite aerogels to impart antibacterial properties, achieving removal of 91.6% and 95.5% for *E. coli* and *S. aureus*, respectively. In addition to a selective adsorption capacity towards organic dyes, GO/montmorillonite aerogels also efficiently removed Cr(VI) ions (equal to 94.9%). The removal of Cr(VI) from water using GO-based aerogels was the focus of various studies, such as those of Li et al. [80], Liang et al. [130], and Wei et al. [135], who managed to eliminate up to about 95%, 99%, and 89% of Cr(VI) ions, respectively. In the last few years, GO-based aerogels have been proven to be excellent absorbent matrices for the removal of other heavy metal ions with genotoxic, mutagenic, and carcinogenic effects on humans and aquatic living organisms, including Pb(II) [121,134], Cu(II) [118], and Fe(III) ions [5]. In addition, the effective elimination of radioactive elements—known as radionuclides—was also proven in the studies of Huo et al. [28] and Lee et al. [29], specifically in the removal of Sr(II) and Cs^+^, employing polyvinyl alcohol (PVA)/GO and polyvinylpyrrolidone (PVP)/GO aerogels, respectively. It is common to involve polymers as crosslinkers and/or stabilizers in the production of GO-based composites. In addition to their specific features—such as water solubility, biodegradability, and nontoxicity—the presence of polymers in the 3D structure can lead to improvements in pollutant adsorption thanks to a synergistic effect with GO. For example, in the study of Huo et al. [28], PVA was selected as the crosslinker due to its ability to form hydrogen bonds with graphene aerogel, which specifically interact with the hydroxyl of PVA molecules. Most of these oxygen-containing functional groups are retained in the composite structures, playing a key role in the hydrophilicity, and as active sites that promote the adsorption of heavy metals and radionuclides. Indeed, it is well known that the presence of active sites in the absorbent materials is decisive for a good adsorption capacity, ensured by both physical and chemical interactions formed between absorbents and adsorbates. For this purpose, other polymers have been employed in the attainment of GO-based aerogels, including polydopamine (PDA) [80,122,136] and polyethylenimine (PEI) [119,133,136]. Xu et al. [136] prepared GO aerogels co-functionalized with PDA and PEI for the adsorption of anionic dyes and organic solvents from wastewater. In addition, to increase the stability of graphene aerogels [122], PDA has a lot of functional groups on the surface (e.g., catechol, amine, and imine). Similarly, PEI is a promising crosslinker that allows an increase in the number of active sites because of its high amine density. As a result, the GO aerogels co-functionalized with PDA and PEI exhibit an efficient adsorption capacity towards methyl orange and amaranth as the model anionic dyes (202.8 mg/g and 196.7 mg/g, respectively), as well as in adsorbing different organic solvents (about 28, 35, 47, 57, and 65 mg/mg for hexane, toluene, dichloromethane, trichloromethane, and tetrachloromethane, respectively).

In general, batch modes are proposed to purify wastewater using GO-based aerogels as adsorbent materials. The pollutant adsorption is usually favored by placing the aerogel in flasks containing the fluid to be treated under magnetic stirring [121,133]. In general, the contaminant concentration in the supernatant is measured at specific time intervals by UV-Vis spectroscopy or high-performance liquid chromatography (HPLC). However, innovative methods have also emerged in recent years; in particular, heterogeneous photocatalysis stands out in permitting the efficient removal of various pollutants using GO-based aerogels [8,129]. Nawaz et al. [8,137] conducted photocatalytic experiments under UV light to remove different drugs—namely, ibuprofen, sulfamethoxazole, and carbamazepine, as a model non-steroidal anti-inflammatory drug (NSAID), antibiotic, and anticonvulsant/antiepileptic compound, respectively. Aerogels based on reduced GO/TiO_2_ were employed, reaching more than 99% photodegradation for all of the contaminants within a time range of 45–90 min. The reduced GO/TiO_2_ composites exhibited a higher photoactivity than the commercial TiO_2_ or a physical mixture of GO and TiO_2_. This outcome is due to several factors—mainly, the chemical bonding between GO and TiO_2_, the interconnected macroporous structure with a large surface area, and many surface sites suitable for anchoring the catalyst. However, the real challenge was faced in the studies of Liu et al. [4] and Deng and Huang [129] in using visible light for the photodegradation of dyes (i.e., methylene blue or rhodamine B) or bisphenol A. 

For this purpose, in both of the studies, aerogels essentially based on GO and silver phosphate (Ag_3_PO_4_) were employed. Indeed, Ag_3_PO_4_ has an auspicious photocatalytic activity under visible light; on the other hand, its low specific surface area and easy photocorrosion limited its application. Hence, Ag_3_PO_4_ and GO aerogels in combination seem to effectively prevent Ag_3_PO_4_ photocorrosion, since GO accelerates charge transfer, being an excellent electron acceptor. In the context of advanced oxidation processes, water purification assisted by heterogeneous Fenton-like reactions [138,139] also emerged as a novel and promising approach. Specifically, Yao et al. [139] proposed a microwave-assisted Fenton reaction to remove rhodamine B. The conventional Fenton process, which uses hydrogen peroxide as the oxidant, has a drawback—namely, a low degradation rate of the organic contaminants in water—leading to the necessity of long process times to assure sufficient removal efficiency. This limit can be overcome using microwave irradiation to shorten the reaction times, specifically generating hot spots on the surfaces of materials with low thermal conductivity. 

Transition metal oxides, such as CuO and Fe_2_O_3_, are good microwave catalysts, capable of absorbing thermal energy from hot spots and producing reactive oxygen species (ROS). For this reason, Yao et al. [139] employed aerogels based on reduced GO and loaded with copper ferrite nanocubes. The aim was to further improve the catalytic activity by exploiting the advantages of reduced GO aerogels. Indeed, a good dispersion of copper ferrite nanocubes on the surface, an enhanced adsorption capacity, rapid transport, and easy access for pollutants within the interconnected open channels of the support to reach the copper ferrite active sites were obtained. These composite aerogels exhibited excellent catalytic performance, with short reaction times, removing up to 95.7% of dye in only 1.0 min.

It is important to point out that several strategies have been attempted to improve the properties and the adsorption performance of GO-based aerogels, such as the incorporation of metallic nanoparticles/nanocrystals [29,123,138,139], or by functionalizing GO with elements such as oxygen, nitrogen, boron, or sulfur [30,110]. In particular, different studies showed that nitrogen could form strong bonds and dope the graphene structure, due to its similar size to carbon atoms, resulting in an increased charge transfer rate on the surface and a better chemical reactivity than pure material [26,30,122,123,130]. Rahmani et al. [26] proposed N-doped reduced GO aerogels for the selective adsorption of oils from wastewater. These aerogels are characterized by a hydrophobic nature, a high specific surface area (340 m^2^/g), and excellent oil adsorption capacities, up to 210 g/g (i.e., the amount of adsorbate per unit weight of adsorbent) in the case of crude oil. In addition, N-doped GO aerogels were found to be very effective in the removal of organic compounds, reaching an adsorption capacity equal to 320 g/g for chloroform. 

Moreover, the adsorbent recyclability was also asserted; indeed, after 10 subsequent cycles, each aerogel maintained 95% of its initial adsorption capacity. For a further enhancement of the performance, metal-based particles were also embedded into N-doped GO aerogels [122,123]. For example, incorporating CoMn_2_O_4_ nanoparticles into the N-doped structure led to a higher degradation rate (namely, 91.3% in 20 min) than the N-doped reduced GO aerogel for removing antibiotics from pharmaceutical wastewater [123]. The rapid drug degradation found in a wide range of pH levels (from 3.0 to 9.0) was attributed to intimate interactions between the dispersed CoMn_2_O_4_ nanoparticles and the N-doped GO network, which promoted an increase in charge transfer and a reduction of the diffusion pathway for the pollutants. On the other hand, Kang et al. [140] proposed applying amino-functionalized GO aerogels to remove quinoline from coking wastewater, i.e., a heterocyclic aromatic organic compound harmful to human health and the environment. The aerogels were prepared via an acid induction method, which promoted the esterification and the amidation of GO and ammonium citrate. This route led to an improvement in the mechanical strength and chemical stability of the aerogels, in addition to the formation of numerous effective adsorption sites. As a result, an adequate adsorption capacity of quinoline was achieved—namely, 103 mg/g. It is worth highlighting that the as-prepared amino-functionalized GO aerogel had a very high specific surface area (up to 736.3 m^2^/g) compared to those obtained in the other studies investigated and reported in Table 4. Specifically, the materials that constituted the GO-based aerogels proposed in each study (mainly considering those published in the last 6 years), the method selected for the aerogel synthesis, its surface area, and the pollutants removed from the water are indicated in Table 4.

## 5. Silica Aerogels

### 5.1. Silica Aerogel Synthesis

Silica aerogels (SAs) show outstanding properties in terms of very high specific surface area (SSA) and porosity coupled with low density and a low dielectric constant. The attainment of aerogels involves a step-by-step process consisting of the preparation of the gel, aging of the gel, and subsequent drying. The gel is prepared via a sol-gel process (preparation of the solution and gelation). At the same time, the drying can be carried out at ambient pressure, in supercritical conditions, or under vacuum [152,153].

During the first step, a precursor (generally a silicon alkoxide) is solubilized in water and mixed with an organic solvent in the presence of a catalyst. The gelation occurs, and the gel is formed from the sol. The final material characteristics, such as the aerogel’s hydrophobicity, will be intensely dependent on the choices made during this step—such as, for example, the percentages of the precursors, type and concentration of the organic solvent, concentration of the catalyst, temperature, and time of the reaction [154].

During the aging step, which generally lasts between 24 h and 5 days, the gel is aged in the mother solution so as to be strengthened. The aging solution concentration and aging time are the parameters that influence the shrinkage, surface area, pore diameter, and pore volume of the final aerogel.

Gel drying is a critical step, which aims to eliminate the liquid contained in the pores. Due to the capillary forces involved, which are very high due to the small size of the pores, the structure can be subject to shrinkage and cracking [155]. Among the various kinds of drying, the first to be proposed has been high-temperature supercritical drying (HTSCD), which consists of inserting the gel together with an organic solvent into a vessel and increasing the temperature (pressure will increase as a consequence). The solution is supercritical because the temperature and pressure are higher than the solvent’s critical values [1]. HTSCD commonly occurs at 18 MPa and 300 °C. Subsequently, low-temperature supercritical drying (LTSCD) has been proposed; this process is based on the use of carbon dioxide as the drying agent, taking advantage of CO_2_’s low critical pressure and temperature values. Common LTSCD operating conditions are 10 MPa and 40 °C [156]. Ambient pressure drying (APD) was developed later; it is based on the chemical modification of the inner surface of the inorganic gel due to derivatization with organosilanes via standard silylation routes [157]. Silylation occurs directly in the aqueous phase of the hydrogel, inducing both the solvent exchange and phase separation of the water and the solvent. A lesser used drying method is based on freeze-drying; in this case, the pore liquid is frozen and sublimed under vacuum in order to prevent the formation of the meniscus between the solid–liquid and liquid–vapor interfaces, obtaining a cryogel [158].

### 5.2. Silica Aerogel Applications

As mentioned before, SAs have unique properties that have made them attractive in many areas [159]. For example, they can be used (1) as absorbents of oils and organic liquids, to control accidental and deliberate releases of these substances during transportation and storage [160]; (2) as humidity sensors and matrices for biosensors [161]; (3) in thermal and acoustic insulation [162,163]; (4) as catalysts, photocatalysts, or catalyst carriers [164,165]; (5) as sorbents to capture CO_2_ gas [166]; (6) in the removal of air pollutants—such as benzene, toluene, ethylbenzene, and xylene (BTEX) [167]—or, in general, of volatile organic compounds (VOCs) [168]; or (7) in wastewater treatments, such as in the removal of dyes [169], or of heavy metal ions [170].

Table 5 lists the main papers in which SAs have been used alone or combined with other materials to remove pollutants from air and water.

Silica aerogels are commonly used to remove volatile organic compounds (VOCs)—such as monocyclic aromatic hydrocarbons (MAHs), polycyclic aromatic hydrocarbons (PAHs), textile dyes, and heavy metals—from wastewater. In some cases, VOCs were removed from waste gas streams. Silica aerogels may also be functionalized or coupled to another material.

Generally, the papers focus on removing a class of pollutants, although Lamy-Mendes et al. [170] used amine-modified silica aerogels to treat different types of contaminants. They focused their study on the removal of two MAHs (benzene and phenol), two dyes (RL and MB), and two metals (copper and lead).

The adsorbents were synthesized through a sol-gel methodology using different sols/gels for various pollutants: (a) in the case of benzene and phenol removal, MTMS was used as the precursor and APTMS as the co-precursor, the aging step lasted 7 days, and the drying occurred at 60 °C for 3 days followed by 3 h at 100 °C; (b) in the case of adsorption of dyes, TMOS was used as the precursor and APTMS as the co-precursor, the aging step lasted 5 days, and the drying occurred at 60 °C for one day followed by 3 h at 100 °C or using supercritical carbon dioxide (scCO_2_); (c) in the case of the removal of metals, MTMS and TEOS were used as the precursors and APTMS as the co-precursor, the aging step lasted 6 days, and the drying occurred in an oven at 60 °C for 3 days or using scCO_2_. The performance of the process was evaluated in terms of mg of adsorbed pollutant per g of aerogel. At the optimized conditions, the authors obtained the removal of 51 mg of benzene, 19 mg of phenol, 44 mg of RL, 15 mg of MB, 124 mg of copper, and 207 mg of lead.

#### 5.2.1. Adsorption of MAHs and PAHs

Different papers have been published on the adsorption of MAHs and PAHs contained in wastewater. For example, Yaqubzadeh et al. removed naphthalene—the smallest PAH with two benzene rings—from a water stream [171]. The sol/gel was prepared using a sodium silicate solution as the Si precursor, TMCS as the surface-modification agent, and isopropanol as the aging solvent. Then, water inside the gel pores was replaced with hexane to lower the capillary forces in the drying stage. The wet gel was left at ambient conditions overnight, and then at 130 °C for 30 min to obtain the aerogel. The obtained hydrophobic aerogel had a specific surface area (SSA) higher than 820 m^2^/g. At the optimized operating conditions in terms of time, initial solution pH, and adsorbent concentration, 73% of naphthalene was removed from the starting solution. Štandeker et al. removed different volatile organic compounds (VOCs)—i.e., toluene, benzene, ethylbenzene, xylene, chlorobenzene, chloroform, 1,2-dichloroethane, and trichloroethylene—from water using SAs with different degrees of hydrophobicity [168]. The gels were prepared using TMOS as the precursor, MTMS and TMES as the methyl groups containing alkoxides, and methanol as the aging solvent. The aerogels were prepared using scCO_2_ at 40 °C and 10 MPa as the drying agent. Depending on MTMS/TMOS or TMES/TMOS molar ratios, SSAs varied from 112 to 872 m^2^/g in the first case, and from 732 to 812 m^2^/g in the second case. Considering an adsorbate concentration of 1 g/L, depending on the VOC, adsorption in the range 0.01–0.13 g/g was obtained. In a subsequent paper, the same authors demonstrated that SAs could also be used for the removal of VOCs from waste gas streams [167]. The aerogels were prepared following the same procedure as in the previous paper; they removed MTEX vapors from the air, packing the adsorbents in a mini-column, through which the flow of air saturated with benzene, toluene, ethylbenzene, or xylene was fluxed. Using the optimized aerogel and two adsorption cycles, they obtained the removal of 1.04 g/g, 1.00 g/g, 0.82 g/g, and 1.07 g/g for benzene, toluene, ethylbenzene, and xylene, respectively.

Yi et al. used hydrophobic/hydrophilic silica aerogels to reduce the concentrations of nitrobenzene, phenol, and methylene blue in wastewater [172]. Hydrophobic SA was prepared using TEOS as the precursor and ethanol as the aging solvent. The drying was conducted at 60 °C for 24 h. For hydrophilic aerogels, a subsequent calcination step at 500 °C for 3 h was necessary. SSAs of the obtained aerogels were of the same order of magnitude; indeed, in the case of the hydrophobic aerogel, SSA was equal to 902 m^2^/g, whereas, in the case of the hydrophilic aerogel, it was 928 m^2^/g. The authors observed that the hydrophobic silica aerogel exhibited higher adsorption capacity on slightly soluble organic compounds (51.8% of nitrobenzene was removed within 1 h, whereas only 9.9% and 17.6% of phenol and MB were removed even after 10 h). Conversely, hydrophilic SA was more effective at adsorbing soluble compounds (the removal ratio of phenol and MB was 57.8% and 64.3%, respectively, within 0.5 h, whereas only 17.8% of nitrobenzene was adsorbed in 1.5 h).

Titania-silica aerogels were prepared vis the sol-gel method to be used in solar-light photocatalysis for the removal of MAHs (*p*-chlorophenol, *p*-nitrophenol, and 4-hydroxybenzoic acid) [173] or PAH (phenanthrene) [174]. In the former paper [173], TEOS and TIOT were used as the precursors of silica and titania aerogels. The co-aging lasted 20 h, and the supercritical drying was conducted at 280 °C and 10 MPa for an hour. The aerogels were then calcinated at 400 °C for 5 h. In correspondence with the optimized aerogels’ photoactivity, the degradations were equal to 86%, 70%, and 95.4% for *p*-chlorophenol, *p*-nitrophenol, and 4-hydroxybenzoic acid, respectively. In the latter paper [174], TiO_2_/SiO_2_ photocatalysts were synthesized and applied for the adsorption and photocatalytic degradation of phenanthrene. Tetrabutyl titanate (TBOT) was used as the precursor of nano-TiO_2_, performing the drying at 80 °C for 4 h. The TiO_2_/SiO_2_ aerogel was calcinated at 400, 600, or 800 °C for 3 h. The aerogel calcined at 800 °C had the best photocatalytic properties, and could degrade phenanthrene completely within 3 h.

Silica aerogel was also coupled with other materials, such as tetrapod-like zinc oxide [175] for the photocatalytic degradation of nitrobenzene, and granulated activated carbon [176] for the adsorption and desorption of benzene.

#### 5.2.2. Adsorption of Dyes

The dyes commonly removed using silica aerogel are acid orange 7 (AO7), Congo red (CR), crystal violet (CV), methylene blue (MB), methyl orange (MO), rhodamine B (RhB), and Rubi Levafix (RL). Silica aerogels were synthesized in different ways by different research groups. Hanu et al. [177] evaluated the effects of various parameters, such as the use of supercritical CO_2_, ultrasound irradiation, and quaternary ammonium salts on the gelation time. The different aerogels obtained were tested for the adsorption of RhB as a model compound for organic water pollutants. The gels were prepared using TEOS and TMOS as the precursors; the samples obtained using TMOS showed a larger surface area than those obtained using TEOS and, consequently, a higher RhB adsorption capacity. Moreover, higher adsorption capacities were obtained in the case of scCO_2_-dried aerogels.

Wei et al. [169] prepared hydrophobic/hydrophilic SAs using TEOS as the precursor and evaluated the effect of pH on the adsorption of four different dyes: RhB, MB, CV, and AO7. The optimum adsorption pH of RhB, MB, CV, and AO7 was 5, 8, 9, and 3, respectively. The removal rate of cationic dyes (RhB, MB, and CV), in correspondence with the optimum pH value, was equal to 90%, 98%, and 90%, respectively. In comparison, the removal rate of anionic dye (AO7) was not more than 30%. Moreover, Han et al. [165] removed cationic dyes from wastewater, comparing the performances of hydrophobic (surface-modified) SA and hydrophilic (hydroxyl-group) SA in terms of adsorption of RhB and MB. They observed that the hydrophobic SA was the best support for removing MB (65.74 mg/g vs. 47.21 mg/g). At the same time, hydrophilic SA gave better results for the adsorption of RhB (185.61 mg/g vs. 134.25 mg/g).

Meng et al. [33] prepared hollow SA fibers engineered based on a wet-spinning approach for dye removal from wastewaters. For the modification of the surfaces, 3-aminopropyl)trimethoxysilane (APTMS) and phenyltrimethoxysilane (PTMS) were used. Moreover, the authors incorporated commercially used photocatalysis-active nanoparticles into SA fibers. The fibers obtained using APTMS as the surface modifier displayed the fastest adsorption for CR, and a removal percentage of 86.3% after 5 min; non-surface-modified SA fibers exhibited the fastest adsorption for MB, and a removal percentage of 98.2% in 5 min. Finally, PTMS-modified SA fibers revealed a high removal speed for both CR and MB (i.e., 62.8% for MB and 80.4% for CR in 5 min).

Yang et al. [178], in order to avoid the use of organosilane reagents—which are commonly used when the drying occurs at ambient pressure—proposed a surface hydroxyl modification method for the synthesis of hydrophilic SA by simply adding metal cations (Ni^2+^, Ba^2+^, Cu^2+^, Fe^3+^, Ca^2+^, and Mg^2+^) during the gelation step. The samples prepared using Ba^2+^ and Mg^2+^ showed better adsorption ability. The best adsorption capacities for RhB and MB reached 2.8 and 40.4 mg/g, respectively.

In two papers, silica-titania aerogels were prepared for the degradation of dyes during photocatalysis. In the first paper [180], a synthesis of a SiO_2_/TiO_2_ binary aerogel was attempted using sodium silicate and titanium tetrachloride as the precursors, using an ambient pressure drying. The chosen model pollutant was methyl orange, and the decolorizing efficiency was equal to 84.9% after 210 min of exposure to UV light irradiation. In the other paper [179], silica-titania gel microspheres were synthesized via the sol-gel process in a W/O emulsion system. The performances in terms of photocatalytic properties of the composite aerogel microspheres were compared to monolithic silica/titania aerogel ones. The two aerogels (microparticles and monolithic) had a similar photocatalytic degradation ratio for methylene blue (about 90% after 3 h). Moreover, the authors observed that there was no or very little change in the degradation ratio among the used and reused SiO_2_/TiO_2_ aerogel microspheres sample, whereas in the case of the second reused monolithic SiO_2_/TiO_2_ aerogel, the catalytic degradation for MB was decreased to about 65%. This result was ascribed to the regular shape and consequent higher recycle ratio of the aerogel prepared in the form of microparticles.

Najafidoust et al. [181] synthesized a BiOI/SA using a sono-solvothermal method, intending to couple the advantages of the specific layered structure of bismuth oxyhalides (BiOX, X = Br, Cl, I) and the high surface area of silica aerogels. Among the different BiOX, BiOI is the most used as a photocatalyst because of its low bandgap (E_g_ = 1.7–1.9 eV). Three organic dyes—MB, AO7, and RhB—were used as model pollutants contained in wastewater. The catalytic performance of the BiOI/SA photocatalyst was measured under solar light, and removal rates of 92.1%, 65.4%, and 22.3% in 120 min were obtained for MB, RhB, and AO7, respectively. In the case of the removal of MB, the influence of the initial dye solution’s pH was evaluated; a pH equal to 9 was found to be optimal, corresponding to a removal of 96.5%.

#### 5.2.3. Adsorption of Heavy Metals

Functionalized silica adsorbents have been used to remove heavy metals, such as copper, lead, cadmium, chromium, nickel, uranium, and zinc. Vareda and Durães [182] adsorbed multiple heavy metals that are contained in watercourses and groundwater. The silica-based, aerogel-like materials were functionalized with mercapto or amine-mercapto groups. The mercapto-functionalized aerogels were prepared using TEOS, MTES, and MPTMS as precursors, while in the case of amine–mercapto-functionalized aerogels, APTMS was also added. The aging time was equal to 5 days, while the drying occurred in an oven (at 60 °C for 48 h and, then, 100 °C for 3 h) to obtain xerogels, or using scCO_2_ to obtain aerogels. The removal percentages of the metals were equal to 39.1% of cadmium, 38.5% of nickel, 39.8% of chromium, and 40% of zinc. In a subsequent paper, Vareda et al. [183] synthesized silica-based aerogels using different nitrogen-containing groups as modifiers—namely, primary amines, secondary amines, urea, and isocyanurate. Depending on the functional group, different precursors and percentages of precursors were used, such as MTES, TEOS, APTMS, AAAPTMS, TTMSI, and UPTMS; the aging time varied from 1 to 6 days, while the drying occurred in an oven at 60 °C for 3 days to obtain xerogels, or using scCO_2_ to obtain aerogels. The removal efficiencies were reported in percentages. Using the best aerogel, a removal of 98.6% of copper, 99.5% of lead, 98.8% of cadmium, and 66.8% of nickel was obtained.

Cadmium removal was also attempted by Shariatinia and Esmaeilzadeh [184], using hybrid silica aerogel (HSA) nanoparticles and two magnetic nanocomposites of HSA with Fe_3_O_4_ nanoparticles and chitosan. TEOS and APTMS were used as the HSA precursors, and the drying occurred at ambient conditions. The Cd^2+^ adsorption was performed by the HSA, chitosan, HAS-Fe_3_O_4_, and HAS-Fe_3_O_4_-chitosan nanocomposite adsorbents in aqueous solutions at different pH values and different adsorbent dosages. The highest adsorption capacities were 58.5, 69.4, 65.8, and 71.9 mg/g for the HSA, chitosan, HAS-Fe_3_O_4_, and HAS-Fe_3_O_4_-chitosan.

Hydrophobic silica aerogel was used in combination with granulated activated carbon to remove uranium from groundwater [185]. The adsorbent was prepared by mixing sol-gel precursors in the presence of granulated activated carbon, gelling the mixture, and supercritically extracting the mixture with methanol.

#### 5.2.4. Adsorption of Other Pollutants

SAs have also been used to remove other pollutants, such as oils [186,187,188] or emerging contaminants [21,189]. Indeed, SAs possess hydrophobicity and oleophilicity and, therefore, can be used to adsorb oil emulsions. For example, Mazrouei-Sebdani et al. [186] prepared SA from sodium silicate precursors via a facile sol-gel method, followed by the low-cost ambient drying process, obtaining outstanding absorption capacity for different oils (up to 4 g of oil/g of aerogel) and eminent absorption recyclability (100%, even after 10 cycles). Abolghasemi Mahani et al. [187] removed crude oil from seawater using MTMS-based aerogels obtained through ambient pressure drying. In correspondence with the optimized conditions, the prepared samples can adsorb heavy and light crude oil to the order of 16.7 and 13.7, respectively.

Hydrophobic aerogels were used by Prasanna et al. [21] to remove pharmaceutical drugs (i.e., doxorubicin, paclitaxel, and diethyl phthalate) from real leachate and hospital wastewater. They used a trimethylsilyloxy-modified silica aerogel as the adsorbent, the adsorption capacity of which—as examined by batch experiments—for doxorubicin, paclitaxel, and diethyl phthalate was 13.80, 14.28, and 17.54 mg/g, respectively.

## 6. Conclusions and Perspectives

Different approaches have been attempted to tune the aerogels’ properties and improve their adsorption performance to remove contaminants from water and air. The studies focused on applying aerogels for air cleaning seem to be in the minority compared to those for wastewater treatment, especially considering some materials such as graphene oxide and chitosan-based aerogels. Hence, it would be worthwhile to carry out further studies attempting to exploit the outstanding properties of these adsorbents for air purification.

Generally speaking, several factors have to be considered in designing the best absorbent for a specific application, including the materials and the composition, the methodology of aerogel synthesis, and the drying technique. In this context, it is essential to highlight that the drying assisted by supercritical CO_2_ has many advantages—mainly the possibility to shorten the drying time (specifically, from days to hours) without collapsing the porous structure. Nevertheless, supercritical drying has been exploited in a limited number of studies compared to freeze-drying. Moreover, a comparison between different drying techniques to obtain aerogels to understand their influence on the pollutant adsorption capacity is missing; such a comparison would provide guidance as to the best drying procedure for this purpose.

A high number of functional groups and a large specific surface area emerged as crucial characteristics of aerogels to effectively remove pollutants. However, it is essential to carefully select materials with different properties depending on the final application, i.e., the contaminants to be removed.

Despite the promising applications of aerogels for removing pollutants, there is still work that must be done to introduce these materials in the adsorbents market. In this context, it is crucial to consider that highly energy-consuming processes are usually required to produce aerogels (i.e., freeze-drying or scCO_2_). Consequently, techno-economic analysis, coupled with kinetic and gelation studies, can be helpful to identify proper reactor configurations and drying times, depending on the material produced. Based on this type of analysis, it would also be possible to optimize experimental conditions and improve the aerogel preparation process in terms of economic viability. This study will facilitate the performance of proper comparisons between presently available adsorbents and aerogels, taking into account costs and adsorption of pollutants, and highlighting the bottlenecks. It is also important to consider that aerogels must also be designed considering recycling times and reuse possibilities.

Future research must also be carried out concerning the use of waste products as raw materials for producing aerogels. Some industries (i.e., paper processing) can be an essential source of cellulose, which, as was mentioned, can be a perfect component to synthesize aerogels with suitable mechanical and adsorption properties. In this sense, this strategy is adequate to reduce costs and introduce a new way to recycle some materials.

Finally, it is important to consider that the particular structure of the aerogels makes possible their surface engineering or functionalization. More studies concerning this issue will be crucial to produce materials able to destroy and/or detect pollutants and improve removal percentages.

## Figures and Tables

**Table 1:** Cellulose-based aerogels for the removal of pollutants. BCA: bacterial cellulose aerogels; NC: nanocellulose; CE: cellulose laurate ester; CNT: carbon nanotubes; NFC: nanofibrillated cellulose; NP: nanoparticles; PANI: polyaniline; RCA: recycled cellulose aerogel; SC: supercritical; SSA: specific surface area; TA: tannic acid.

Materials	Process	Characteristics	Application	Ref.
NFC + amine	Freeze-drying	SSA: 7.1 m^2^/g Adsorb: 1.39 mmol CO_2_/g	CO_2_ adsorbent	[46]
NFC + CNT + metal NP	Freeze-drying	SSA: 10 m^2^/g Adsorb: 97–100%	Gas adsorbent	[6]
BCA modified	Freeze-drying	SSA > 169 m^2^/g Adsorb: up to 185 g/g	Oil and solvent removal	[47]
BCA + SiO_2_	Freeze-drying	Superleastic high quality adsorption (up to 14)	Oil and solvent removal	[25]
RCA	Freeze-drying	Adsorb: 49–95 g/g	Oil removal	[48]
RCA	Freeze-drying	Porosity: 97.3% Adsorb: 13.9–24.4 g/g	Oil removal	[49]
RCA	Freeze-drying	Porosity: 98.7% Adsorb: 34.5 g/g	Oil removal	[50]
NC	Freeze-drying	Porosity: 98% SSA: 85–147 m^2^/g Adsorb: 45–127 mg/g	Dye and metal removal	[51]
NC	Freeze-drying	Adsorb: 90% vol/vol 20–40% (wt/wt)	Oil removal	[52]
NC	Freeze-drying	Porosity > 99% SSA: 95–196 m^2^/g Adsorb: 159–260 mg/g	Oil and solvent removal	[24]
NC	Freeze-drying	Porosity > 99% SSA: 11 m^2^/g Adsorb: 200–375 g/g	Oil and solvent removal	[53]
NC	Freeze-drying	Adsorb: 150 mg/g	Dye removal	[54]
NFC + GO	Lyophilization	SSA: 128–581 m^2^/g Versatile adsorption	Oil and dye removal	[55]
NFC + PANI	Freeze-drying	Adsorb: 600 mg/g 1363 mg/g	Dye removal	[22]
CE	Freeze-drying SC-drying	SSA: 105–152 m^2^/g Adsorb: 9.63–18.38 mmol/g	Dye removal	[56]
NFC + TA	Freeze-drying	SSA: 76–151 m^2^/g Adsorb: up to 108 g/g	Solvent removal	[57]

**Table 2:** Application of chitosan-based aerogels. β-CD: β-cyclodextrin; AO7: acid orange 7; ARS: alizarin red S; CMC: carboxymethyl cellulose; CMCh: carboxymethyl chitosan; CR: Congo red; CV: crystal violet; GO: graphene oxide; MB: methylene blue; MO: methyl orange; MOF: metal-organic framework; PDA: polydopamine; PDMS: polydimethylsiloxane; PVA: polyvinyl alcohol; RhB: rhodamine B; SSA: specific surface area.

Materials	Characteristics SSA (m^2^/g)	Aerogel Preparation	Improvement	Ref.
Alginate/Chitosan/Melamine	SSA: - Pb(II) (1331.6 mg/g)	Alginate crosslinking with CaCl_2_; freeze-drying	Complexation of alginate; improve adsorption due to amino groups	[27]
Chitosan/Bacterial Cellulose/MOF	SSA: 268.7 Cu^2+^ (206.6 mg/g) Cr^6+^ (152.1 mg/g) Organic Dye (almost 100% adsorption)	Hydrogels by mixing bacterial cellulose and chitosan; freeze-drying	Bacterial cellulose enhanced mechanical properties; MOF increases the surface area	[67]
Chitosan/PDA	SSA: 77.3 (freeze-drying), SSA: 4.3 (vacuum-drying) Cr(VI) (374.4 mg/g) Pb(II) (441.2 mg/g)	Crosslinking with glutaraldehyde; freeze- or vacuum-drying	Adsorption improvement; enhanced stability and acidic water resistance	[68]
Chitosan/Waste Paper Office	SSA: - Cu(II) (156.3 mg/g)	Hydrogel with NaOH and urea; freeze-drying	Increasing mechanical strength and adsorption capacity	[69]
β-CD/Chitosan/ Hexamethylenetetramine	SSA: - Cr(VI) (333.8 mg/g) MB (395.7 mg/g) RhB (364.3 mg/g) ARS (261.0 mg/g) AO7 (134.1 mg/g)	Physical via pH modification and glutaraldehyde crosslinking; freeze-drying	Stability and adsorption properties	[70]
Chitosan/Carboxylated Carbon Nanotubes	SSA: 106.4 U(VI) (307.5 mg/g)	Crosslinking with epichlorohydrin; freeze-drying	Improve mechanical strength and adsorption of uranyl; improve the CNT dispersion	[71]
Chitosan/Cellulose Sulfate	SSA: 0.78 Pb(II) (137.8 mg/g)	Crosslinking with glutaraldehyde; freeze-drying	No problems with cellulose sulfate particles cohesiveness	[72]
Chitosan/GO	SSA: 345 Cu(II) (25.4 mg/g)	Hydrogel formation; freeze-drying	Improve GO removal and compound adsorption	[73]
Chitosan/GO	SSA: - Dye (430.99 mg/g)	Hydrogel formation; ice templating and freeze-drying	Improve GO removal and compound adsorption	[61]
Chitosan/GO	SSA: - Indigo Carmine (534.4 mg/g) MB (168.6 mg/g)	Crosslinking with glutaraldehyde; freeze-drying at different times	Improve dye adsorption and increase the mechanical strength	[74]
Chitosan Grafted with GO	SSA: 33 CO_2_ (0.26 mmol/g)	Physical gelation; freeze-drying	Improve CO_2_ capture	[75]
Chitosan/Reduced GO/Silica/PDMS	SSA: - Oil/Water separation (18.45 g/g)	Hydrogel formation; directional freeze-drying	Hydrophobic properties; thermal stability; mechanical resistance	[7]
Chitosan	SSA: - Cu(II) (108.14 mg/g) Pb(II) (143.73 mg/g) Cd(II) (84.62 mg/g)	Immersion method after producing the aerogel; freeze-drying	Improving adsorption	[66]
Chitosan/GO/Lignosulphonate	SSA: 74.8 MB (1023.9 mg/g)	Hydrogel formation; freeze-drying	Improve adsorption and regeneration	[76]
Chitosan/Microalgae Biomass	SSA: 6.48 U(VI) (571 mg/g)	Hydrogel formation; dried at room temperature	Possible biomass separation.	[77]
Chitosan/Cellulose/MOF	SSA: 457.75 MB (526.3 mg/g)	Crosslinking with epichlorohydrin; freeze-drying	Improve mechanical resistance and SSA	[65]
Chitosan/Cellulose Nanofibers	SSA: - Oil/Water separation (253.3 g/g)	Crosslinking with glutaraldehyde; directional freeze-drying	Improve mechanical resistance and anisotropic thermal insulation properties	[78]
Chitosan/Cellulose Nanofibers	SSA: - Pb(II) (252.6 mg/g)	Hydrogel formation; directional freeze-drying	Improve adsorption	[62]
Chitosan/Polybenzoxazyne/Sodium Montmorillonite	SSA: -	Gelation with benzoxazine polymer; freeze-drying	Subsequent ring polymerization to increase thermal stability and hydrophilicity	[79]
Chitosan/PDA/GO	SSA: - Cr(VI) (312.05 mg/g)	Gelation due to the interactions; freeze-drying	Increase adsorption due to the addition of active adsorption sites	[80]
Chitosan/Silica	SSA: 237.4 Cd(II) (98.49%)	Sol–gel technique; drying at 80 °C	Increase surface area	[81]
Chitosan/Soot	SSA: 11 MB (250 mg/g) Indigo Carmine (275 mg/g) Naphthalene (7 mg/g)	Crosslinking with glutaraldehyde; freeze-drying	Increase mechanical resistance; reduce adsorption of anionic dyes but increase adsorption of cationic dyes; improve soot dispersion	[82]
Chitosan/titanium	SSA: - Cr(VI) (171 mg/g)	Metal-binding and crosslinking with glutaraldehyde; drying at 40 °C	Improve adsorption; mechanism of adsorption, reduction, and re-adsorption	[83]
Chitosan/Lanthanum Hydroxide	SSA: 172.74 Phosphorus (148.33 mg/g)	Hydrogel formation; ScCO_2_	Increase phosphorus adsorption	[84]
CMCh/Magnetite/PDA	SSA: 106.7 MB (217.43 mg/g) CV (262.27 mg/g) MO (83.47 mg/g) CR (92.83 mg/g)	Crosslinking with glutaraldehyde; freeze-drying	Increase water solubility; possible magnetic separation; improve adsorption and avoid aggregation of magnetic particles	[63]
CMCh/GO	SSA: - Ag(I) (151.30 mg/g) Pb(II) (249.38 mg/g) Cu(II) (95.37 mg/g)	Crosslinking with TPP or glutaraldehyde; freeze-drying	Increase mechanical resistance and prevent GO stacking and increase adsorption	[85]
CMCh /Nanobentonite/Oxidized Cellulose	SSA: - Cr(VI) (98.90%) Co(III) (97.45%) Cu(II) (99.01%)	Hydrogel formation; freeze-drying	Increase stability and adsorption efficiency	[86]
Reduced GO/Chitosan/PDA	SSA: 51.76 Oil/Water separation (adsorption efficiency between 90 and 97%)	Crosslinking with glutaraldehyde; freeze-drying	Superhydrophobic and stable aerogel under water	[87]
Silylated Chitosan	SSA: 27.10 Oil/Water sep. (63 g/g)	Hydrogel formation; directional freeze-drying	Superhydrophobic aerogel with enhanced mechanical resistance	[88]
CMCh /Nanocellulose	SSA: - MB (785 mg/g)	Crosslinking between the materials; freeze-drying	Increase adsorption	[89]
CMCh/Gallic Acid/Fe^III^	SSA: - Pb(II) (97.15 mg/g) Cd(II) (99.75 mg/g) Cu(II) (98.50 mg/g)	Crosslinking, electrostatic interactions, and metal coordination; freeze-drying	Mechanical resistance and increase adsorption	[90]
Chitosan/Zeolite	SSA: 561 CO_2_ (4.23 mmol/g)	Hydrogel formation; freeze-drying	Increase surface area and mechanical resistance; improve CO_2_ capture, and it is possible to reuse it.	[91]
Chitosan/Agarose with Fe and Al Nanocomposites	SSA: 384.14 Dyes (95% rejection) As(V) (102.45 mg/g) F (81.56 mg/g)	Hydrogel formation; freeze-drying	Increase aerogel stability due to the addition of agarose; increase As (V) and F removal due to the addition of Fe and Al	[92]
Chitosan/Agarose	SSA: - Oil/Water separation (99% pure water)	Crosslinking with genipin; freeze-drying	Increase macroporosity as well as stability due to the addition of agarose	[93]
Chitosan with Anchored MoS_2_	SSA: 32.46 Gold recovery by gold reduction from Au(I) to Au(0)	Crosslinking with glutaraldehyde; freeze-drying	Improve thiosulfate leaching, and can produce metal ion photoreduction; increase mechanical resistance	[94]
Chitosan/GO/CMC	SSA: - Organic dye (3190 mg/g) Cr(VI) (127.4 mg/g)	Crosslinking between the polymer functional groups; freeze-drying	Increase GO water stability by protecting it with a core-shell structure (chitosan as shell and CMC and GO in the core)	[95]
Chitosan/GO	SSA: - Pb(II) (747.5 mg/g) Cr(VI) (292.8 mg/g) Cr(VI) (457.5 mg/g) MB (584.6 mg/g) RhB (492.8 mg/g) MO (189.4 mg/g) Eosin Y (124.8 mg/g) Phenol (73.1 mg/g)	Crosslinking with glutaraldehyde; electrospraying with freeze-drying	Structure with microchannels to achieve a faster máximum equilibrium adsorption rate.	[96]
Chitosan/Cellulose Nanofibers	SSA: 315.10 Anionic dye (1428.7 mg/g)	Crosslinking with epichlorohydrin; freeze-drying	Increase surface area and adsorption capacity.	[23]
Quaternized Chitosan/PVA	SSA: - CO_2_ capture (4.23 mmol/g)	Crosslinking with PVA and glutaraldehyde; freeze-drying	Quaternary ammonium groups for moisture swing CO_2_ capture; increase CO_2_ capture by reducing the adsorption half-time.	[64]
Chitosan Aerogel coated with Chitosan Hydrogel	SSA: - Oil/Water separation (oil purity 99.8%)	Hydrogel formation; freeze-drying	Superhydrophilicity and superoleophobicity	[97]
Chitosan/GO/PDA	SSA: - U(VI) (415.9 mg/g)	Electrostatic interactions; freeze-drying	Improve GO recovery and increase U(VI) adsorption due to the PDA functional groups	[98]
Succinyl Chitosan/Sodium Alginate	SSA: - Oil/Water separation (99% efficiency)	The obtained aerogel was crosslinked with calcium chloride/aluminum chloride, and then with glutaraldehyde; freeze-drying	Improve chitosan water solubility (succinyl chitosan); improved mechanical properties due to the preparation technique	[99]
Sulfhydril Chitosan/Sodium Alginate	SSA: - Cu(II) (81.15 mg/g) Pb(II) (38.87 mg/g) Cd(II) (38.15 mg/g) MO (57.75 mg/L) MB (51.62 mg/L) RhB (58.65 mg/L) Sudan I (48.37 mg/L)	Crosslinking with glutaraldehyde; freeze-drying	Increase of adsorption capacity for different compounds	[100]
Chitosan/GO	SSA: 641.6 Oil/Water separation (Oil 12.45 g/g)	Crosslinking with glutaraldehyde; freeze-drying	Decrease water adsorption	[101]
Chitosan/PDMS/Magnetite	SSA: - Oil/Water separation (Oil 22.38 g/g)	Hydrogel formation; freeze-drying and dip-coating	Increase hydrophobicity; magnetic properties	[102]
CMCh/GO/PDA	SSA: - Cu(II) (170.3 mg/g) Ni(II) (186.8 mg/g) Pb(II) (312.8 mg/g)	Use of PDA as a crosslinker with the GO and self-assembled with the CMCh; freeze-drying	Increase mechanical resistance and improve recovery of the material; increase water solubility and stability as well as adsorption of pollutants.	[103]

**Table 3:** Advantages and disadvantages of GO-based aerogels.

PROS	CONS
Lightness Very high surface area Good mechanical and thermal resistance Excellent electrical conductivity Properties can be improved/tailored by connecting functional groups High adsorption capacity Ease of large-scale production and reproducibility	Relatively expensive production Long production times Brittleness GO’s inherent drawbacks, such as complex structure and random distribution of oxygen moieties on the surface

**Table 4:** Graphene oxide-based aerogels for water treatment. 4-NP: 4-nonylphenol; Ce_2_O_3_: cerium oxide; CMC: carboxymethyl cellulose; CR: Congo red; DMPDA: dimethylaminopropylamine; DMAC: dimethylacetamide; DMF: N,N-dimethylformamide; DMSO: dimethyl sulfoxide; EDA: ethylenediamine; GO: graphene oxide; IPEDA: N-isopropylethylenediamine; MB: methylene blue; MO: methyl orange; M1180: mineral oil; MMT: montmorillonite; Nd_2_O_3_: neodymium oxide; NPs: nanoparticles; PB: Prussian blue; PDA: polydopamine; PEI: polyethylenimine; Pr_2_O_3_: praseodymium oxide; PVA: polyvinyl alcohol; PVP: polyvinylpyrrolidone; QAS: quaternary ammonium salts; rGO: reduced graphene oxide; RhB: rhodamine B; SA: sodium alginate; SSA: specific surface area; TiO_2_: titanium oxide; ZIF-67: zeolitic imidazolate framework-67.

Aerogel Materials	Preparation Technique	SSA (m^2^/g)	Pollutant/Application	Ref.
GO/PEI	Simple stirring, freeze-drying	61.9	Mercury in natural waters	[133]
GO/Aminated Lignin	Modified Hummers’ method, sonication, UV-initiation, freeze-drying	8.1–16.1	Malachite green (a fishery dye) in wastewater	[128]
Peanut Shell/GO	Modified Hummers’ method, sonication, freeze-drying	12.1–64.2	Norfloxacin (an antibiotic) in wastewater	[20]
Ag_3_PO_4_/GO	Modified Hummers’ method, sonication, freeze-drying	125.6–169.4	MB dye in wastewater	[129]
rGO	Hydrothermal reduction assembly method	136.7	Pb(II) ions in wastewater	[121]
PVA/GO	Crosslinking method, freeze-drying	*-*	Sr(II) (a radionuclide) in wastewater	[28]
Chitosan/GO	Hummers’ method, crosslinking method, freeze-drying	4.85	4-NP (an estrogen-mimicking compound) in wastewater	[3]
Lysine/EDA/GO	Double crosslinking, chemical reduction method	-	MB dye in wastewater	[117]
SA/Gelatin/GO	“Hydrophilic assembly-sustained release gelation” two-step method	-	MB and CR dyes in wastewater	[141]
DMPDA/IPEDA/GO	Hydrothermal method, cross-linking, freeze-drying	-	Cu(II) ions in wastewater	[118]
Amino-Functionalized GO	Acid induction method	667.4–736.3	Quinoline in coking wastewater	[140]
Chitosan/Gelatin/GO	Embedding technique, freeze-drying, dehydrothermal treatment	-	Microcontaminants in drinking water; antibiotics (ofloxacin, ciprofloxacin), Pb(II) ions	[134]
PB NPs into PVP/rGO	γ-Irradiation	-	Dyes (MB), oils (n-hexadecane), radionuclides (Cs^+^ ions) in wastewater	[29]
GO	Modified Hummers’ method, sonication, freeze-drying	16.9–330.7	Fe(III) ions in wastewater	[5]
Chitosan/PDA/GO	modified Hummers’ method, crosslinking, freeze-drying	-	Cr(VI) ions in wastewater	[80]
N-Doped GO	Hydrothermal self-assembly method, freeze-drying	-	Cr(VI) ions in wastewater	[130]
Silver Phosphate/GO	Hummers’ method, electro-spraying, freeze-drying	-	RhB and bisphenol A in wastewater	[4]
rGO-TiO_2_/SA	Hydrothermal method, freeze-drying	-	Drugs (ibuprofen, sulfamethoxazole) in wastewater	[8]
rGO/TiO_2_	Sonication, hydrothermal method, freeze-drying	65.0–209.0	Carbamazepine (a drug) in wastewater	[137]
GO/QAS	Modified Hummers’ method, “spray-penetration-flocculation” method, freeze-drying	-	Dyes (MB), organic solvents (toluene, n-dodecane, cyclohexane, hexane, petroleum ether, dichloromethane, chloroform), oils (gasoline, soybean oil), bacteria (*S. aureus*, *E. coli*) in wastewater	[131]
N-Doped rGO	Modified Hummers’ method, hydrothermal and thermal annealing methods	340.0	Oils, organic solvents in wastewater	[26]
CoFe_2_O_4_ into N-Doped PDA/rGO	Ultrasound, hydrothermal method, freeze-drying	97.7	Tetracycline (an antibiotic) in wastewater	[122]
CoMn_2_O_4_ NPs into N-Doped rGO	Hummers’ method, hydrothermal method, freeze-drying	-	Oxytetracycline (an antibiotic) in wastewater	[123]
GO	Modified Hummers’ method, chemical reduction method	211.5	Dyes (MB, Acid Red 88, Orange II) in wastewater	[109]
GO-MMT/SA	Crosslinking, freeze-drying	85.2–266.3	Dyes (MB, RhB) in wastewater	[126]
rGO/Silica	Modified Hummers’ method, sol-gel method	391.0–596.0	Oils (M1180, oil blue N) in wastewater	[125]
Nickel Alginate/GO	Modified Hummers’ method, ionic gelation method, freeze-drying	5.1	MB dye in wastewater	[142]
Phytic Acid/GO	Modified Hummers’ method, hydrothermal method, freeze-drying	41.8–258.1	Uranium in water	[143]
rGO	Improved Hummers’ method, gelation, freeze-drying	-	Microorganisms in activated sludge and dyes (MB, CR, MO) in wastewater	[144]
Fe_3_C NPs into rGO	Hydrothermal synthesis, freeze-drying, high-temperature treatment	324.8	MO dye in wastewater	[138]
GO/Nanocellulose	Modified Hummers’ method, blending, freeze-drying	18.0–35.0	MB dye and tetracycline (drug) in wastewater	[145]
Biochar/rGO	Ultrasonication, hydrothermal reduction, freeze-drying	-	Cr(VI) ions in wastewater	[135]
GO/Alkali Lignin	Hummers’ method, ultrasonication, lyophilization	24.8–41.5	MB dye in wastewater	[146]
rGO	Modified Hummers’ method, hydrothermal method, freeze-drying	-	Mercury and fluoride in wastewater	[147]
CMC/rGO	Hydrothermal method	-	Organic solvents (acetone, DMAC, DMF, DMSO, methanol, ethanol) and dyes (RhB) in wastewater	[132]
Fe_3_O_4_ Nanoaggregates_/_ Cellulose/GO	Improved Hummers’ method, self-assembled gelation	-	Dyes (CR, MB) and metal ions (Cu^2+^, Pb^2+^, Cd^2+^, Cr^3+^) in wastewater	[148]
PDA/ PEI/GO	Modified Hummers’ method dopamine self-polymerization, ultrasound, freeze-drying	-	Dyes (MO, Amaranth) and organic solvents (hexane, toluene, dichloromethane, trichloromethane, tetrachloromethane) in wastewater	[136]
GO/Lignosulfonate	Hummers’ method, sonication, freeze-drying	74.8	MB dye in wastewater	[76]
rGO/ZIF-67	Modified Hummers’ method, in situ assembly of ZIF-67, freeze-drying	70.0–491.0	Dyes (crystal violet and MO) in wastewater	[149]
Copper Ferrite/rGO	Hummers’ method, hydrothermal method, freeze-drying	11.4	RhB dye in wastewater	[139]
rGO/Montmorillonite	Modified Hummers’ method, sol-gel method, freeze-drying	-	MB dye, Cr(VI) ions, bacteria (*S. aureus*, *E. coli*) in wastewater	[124]
GO, rGO/Nd_2_O_3_, rGO/Pr_2_O_3_, rGO/Ce_2_O_3_	Hydrothermal method, freeze-drying	57.3–385.2	RhB dye in wastewater	[150]
GO/PEI	Sol-gel method, freeze-drying	453.4–599.8	MO and MB dyes in wastewater	[119]
CMC/rGO	Ultrasonication, crosslinking, freeze-drying reduction	-	MB dye in wastewater	[151]

**Table 5:** AO7: acid orange 7; APTMS: (3-aminopropyl)trimethoxysilane; AAAPTMS: N1-(3-trimethoxysilylpropyl)diethylene triamine; BiOX (X = Cl, Br, I): bismuth oxyhalides; BNP: bismuth nitrate pentahydrate; CR: Congo red; CV: crystal violet; MAHs: monoaromatic hydrocarbons; DVTHP: 2,5-divinyltrimethoxysilanethiophene; HMDZ: hexamethyldisilazane; MB: methylene blue; MO: methyl orange; MPTMS: 3-mercaptopropyl)trimethoxysilane; MTES: methyltriethoxysilane; MTMS: methyltrimethoxysilane; NaSi: sodium silicate; NPs: nanoparticles; PAHs: polycyclic aromatic hydrocarbons; RhB: rhodamine B; RL: Rubi Levafix; SA: silica aerogel; SSA: specific surface area; TBOT: tetrabutyl titanate; TEOS: tetraethyl orthosilicate; TIOT: tetraisopropyl orthotitanate; TIS: titanyl sulfate; TMCS: trimethylchlorosilane; TMES: trimethylethoxysilane; TMOS: tetramethyl orthosilicate; TTMSI: tris[3-(trimethoxysilyl)propyl]isocyanurate; T-ZnO: tetrapod-like zinc oxide; UPTMS: 1-[3-(trimethoxysilyl)propyl]-urea; VOCs: volatile organic compounds; WW: wastewater.

Material	Preparation Technique	Precursor/Surface Modification Agent	SSA (m^2^/g)	Pollutant	Ref.
*Adsorption of MAHs and PAHs*					
SA	Sol-gel process + ambient drying and then 130 °C	NaSi and TMCS	823	Naphthalene from WW	[171]
SA	Sol-gel process + supercritical drying	TMOS, MTMS, and TMES	112–872	VOCs from WW	[168]
SA	Sol-gel process + supercritical drying	TMOS, MTMS, and TMES	112–812	BTEX from air	[167]
SA	Sol-gel process + ambient pressure drying at 60 °C	TEOS	902–928	Nitrobenzene, phenol and MB from WW	[172]
Amine-Functionalized SA	Sol-gel process + drying at 60 °C and then 100 °C	MTMS and APTMS	72–458	Benzene and phenol	[170]
SA/TiO_2_	Sol-gel process + supercritical drying	TEOS and TIOT	320–357	*p*-chlorophenol, *p*-nitrophenol, 4-hydroxybenzoic acid	[173]
SA/TiO_2_	Sol-gel process + oven drying	TBOT	424–644	Phenanthrene	[174]
SA/T-ZnO	Sol-gel process + drying at 60 °C	TEOS and TMCS	86.8	Nitrobenzene	[175]
Carbon-SA	Sol-gel process + ambient pressure oven drying	Cheap water glass	710–758	Benzene	[176]
*Adsorption of dyes*					
SA	Sol-gel process + oven drying at 60 °C and then 100 °C or supercritical drying	TMOS and APTMS	191–817	RL and MB	[170]
SA	Sol-gel process + ambient pressure oven drying at 70 °C or supercritical drying	TMOS and TEOS	581–731	RhB	[177]
SA	Sol-gel process + ambient pressure drying at 60 °C and then 100 °C	TEOS	468–855	MB and RhB	[178]
SA	Sol-gel process + ambient pressure oven drying at 60 °C and then 180 °C	TEOS	889	MB, CV, AO7, and RhB	[169]
SA	Sol-gel process + ambient pressure drying	TEOS and HMDZ	629–880	MB and RhB	[165]
SA	Sol-gel process + vacuum oven drying at 80 °C	NaSi	736	MB and CR	[33]
SA/TiO_2_	Sol-gel process + ambient pressure drying at 70 °C	TIS	415	MB	[179]
SA/TiO_2_	Sol-gel process + ambient pressure drying at 60 °C	TiCl_4_ and TMCS	605	MO	[180]
BiOI/SA	Sono-solvothermal method	BNP and TEOS	206	MB, AO7, and RhB	[181]
*Adsorption of heavy metals*					
SA	Sol-gel process + oven drying at 60 °C or supercritical drying	MTES, TEOS, and APTMS	28–759	Copper and lead	[170]
Mercapto and Amine-Mercapto-Functionalized SA	Sol-gel process + oven drying at 60 °C and then 100 °C or supercritical drying	TEOS, MTES, MPTMS, and APTMS	Up to 702	Copper, lead, chromium, cadmium, nickel, and zinc	[182]
Amines, Urea, and Isocyanurate-Functionalized SA	Sol-gel process + ambient pressure oven drying at 60 °C or supercritical drying	MTES, TEOS, APTMS, AAAPTMS, TTMSI, and UPTMS	3–1006	Copper, lead, cadmium, and nickel	[183]
SA, SA/Fe_3_O_4_, SA/Fe_3_O_4_/Chitosan	Sol-gel process + ambient pressure drying at 50 °C	TEOS and APTMS	/	Cadmium	[184]
Carbon-SA	Sol-gel process + supercritical drying	TMOS	579–978	Uranium	[185]
*Adsorption of other substances*					
SA	Sol-gel process + ambient pressure oven drying at 80 °C and then 200 °C	TMCS	870	Oil	[186]
SA	Sol-gel process + ambient pressure oven drying at 80 °C, then 100 °C and then 180 °C	MTMS	447–712	Crude oil	[187]
Thiophene-Bridged SA	Sol-gel process + ambient pressure drying	TEOS and DVTHP	834	Benzene and oil	[188]
Trimethylsilyloxy-Modified SA	/	/	729	Doxorubicin, paclitaxel, diethyl phthalate, and RhB	[21]
SA/Basalt Fibers Filled into a PEEK Tube	Sol-gel process + drying at 80 °C	APTMS	/	Estrogens	[189]

## Data Availability

The data presented in this study are available on request from the corresponding author.

## Sample Availability

Samples of the compounds are not available from the authors.
